# Supplementary material for: Bacterial expression, purification and folding of exceptionally hydrophobic and essential protein: Surfactant Protein-B (SP-B)
Source: PLoS One. 2025 Apr 25;20(4):e0321446. doi: 10.1371/journal.pone.0321446 (PMC12027065; doi:10.1371/journal.pone.0321446)
Supplement: S1 Fig — The signal at 222 nm, which is sensitive to α-helical structure decreases with increasing TCEP. The spectra have been normalized by relative protein concentration. (DOCX) [file pone.0321446.s001.docx]

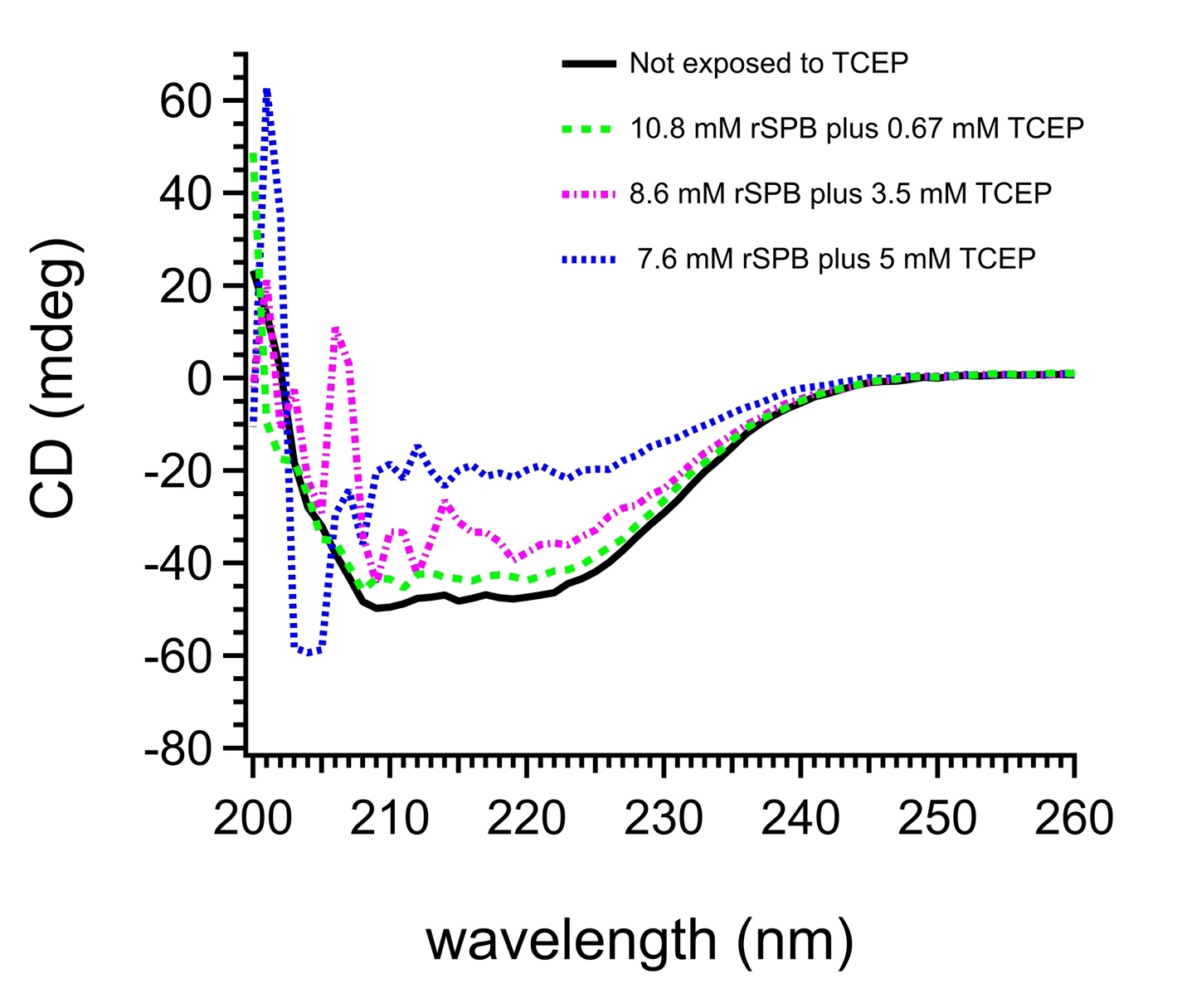


**S1 Figure.** The CD spectra of rSP-B in methanol and increasing concentrations of reducing agent Tris 2-carboxyethyl phosphine (TCEP). The signal at 222 nm, which is sensitive to α-helical structure decreases with increasing TCEP. The spectra have been normalized by relative protein concentration.
