# Supplementary material for: Bacterial expression, purification and folding of exceptionally hydrophobic and essential protein: Surfactant Protein-B (SP-B)
Source: PLoS One. 2025 Apr 25;20(4):e0321446. doi: 10.1371/journal.pone.0321446 (PMC12027065; doi:10.1371/journal.pone.0321446)
Supplement: S6 Data — (PDF) [file pone.0321446.s006.pdf]

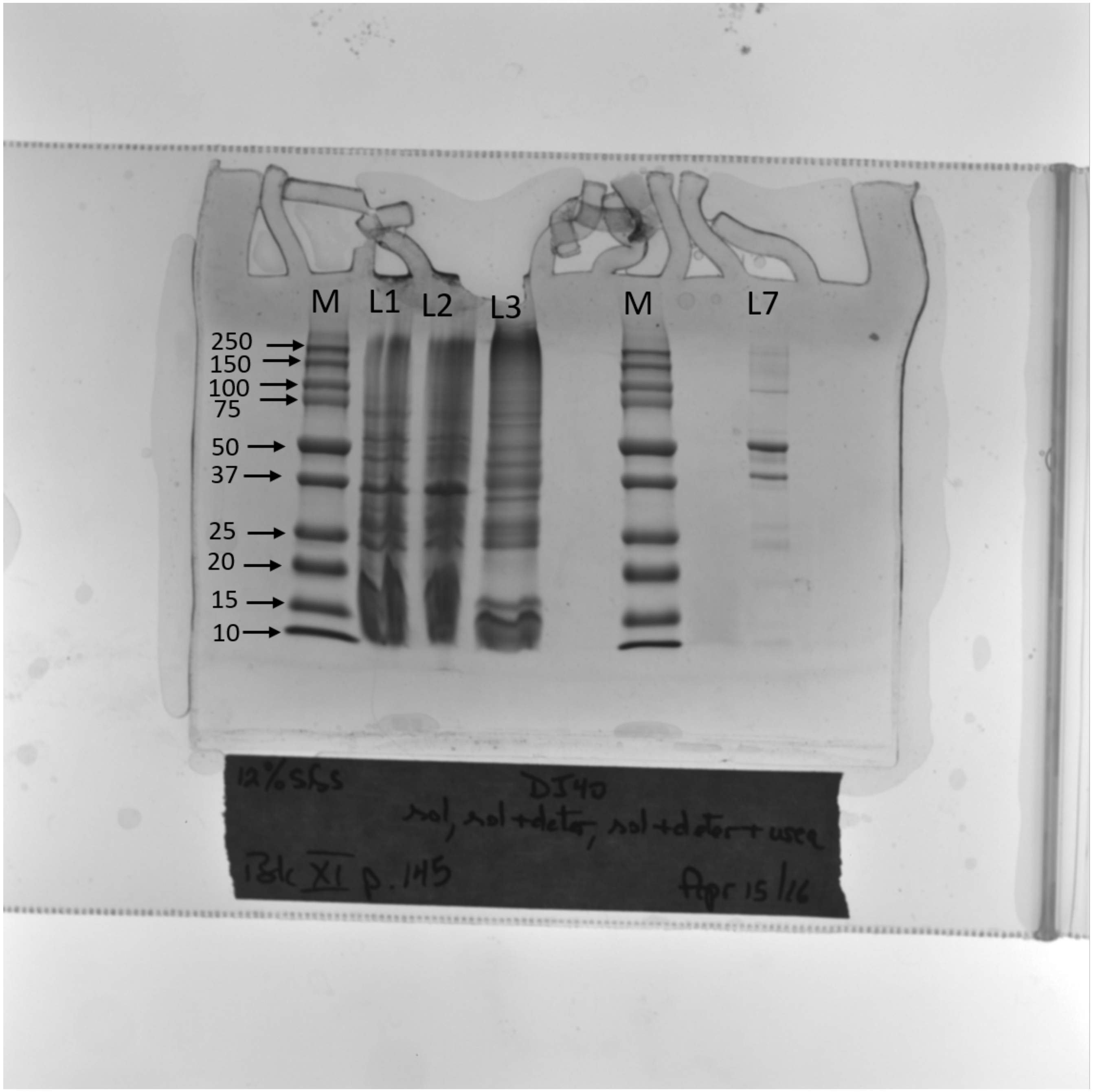

Figure 3A

M = marker

L1 = cell lysate in 1XTBS

L2 = cell lysate in 1XTBS and CHAPS

L3 = lysate in TBS, CHAPS and Urea

L7 = BSA control

Blot duplicate of Fig 3A

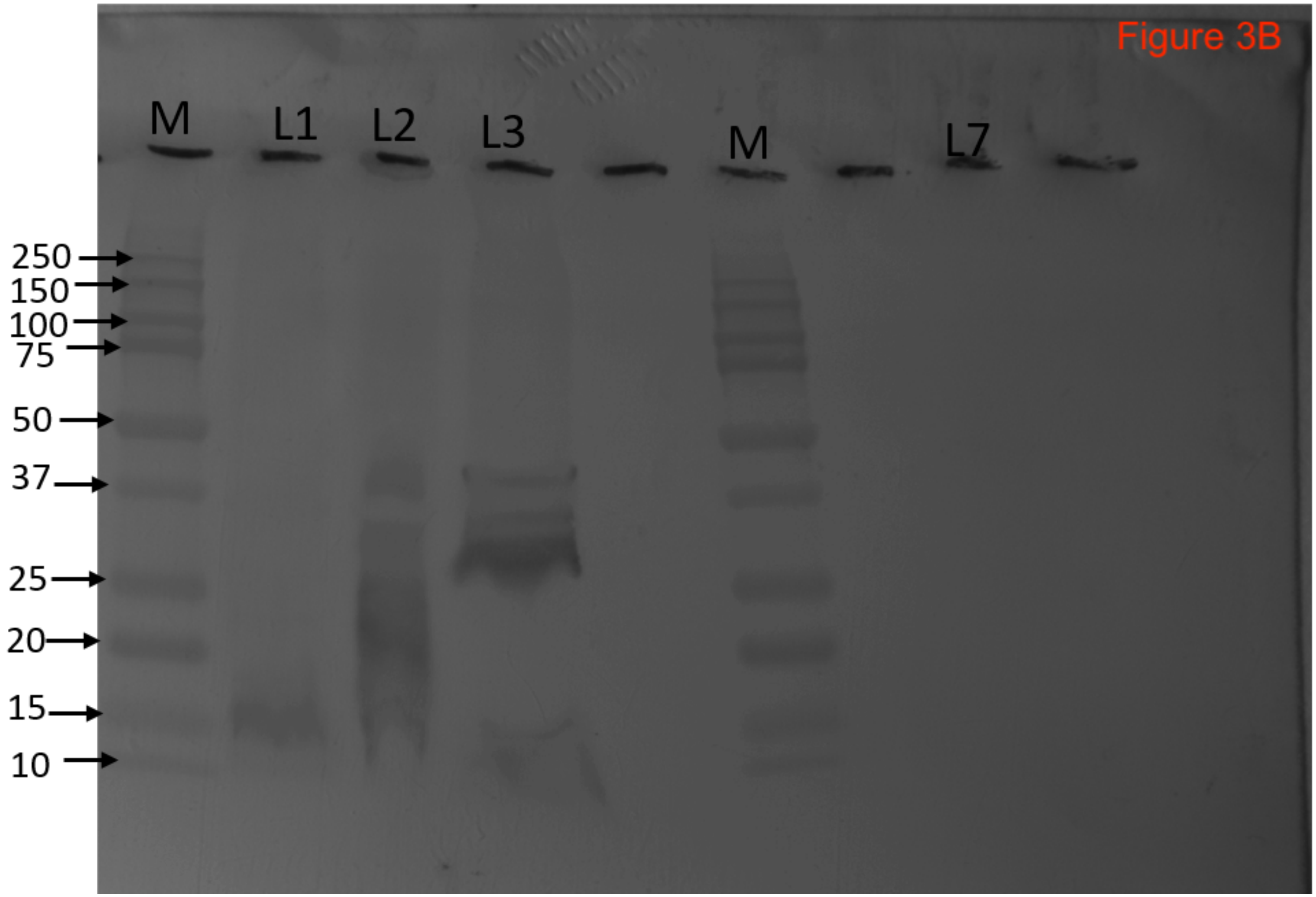

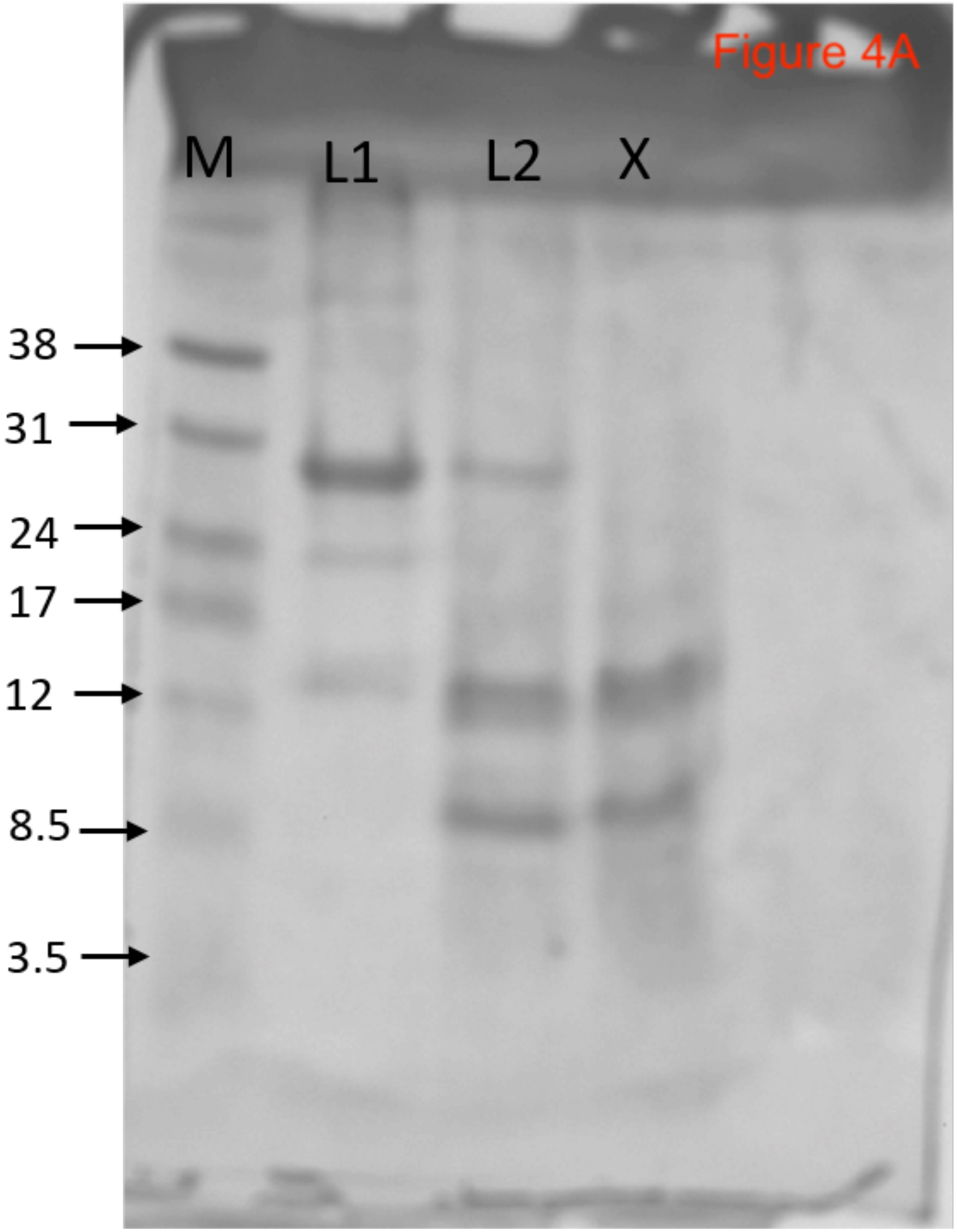

M = marker
L1 = SN-SP-B fusion protein
L2 = digest (SN and SP-B)
X = duplicate sample (cut off)

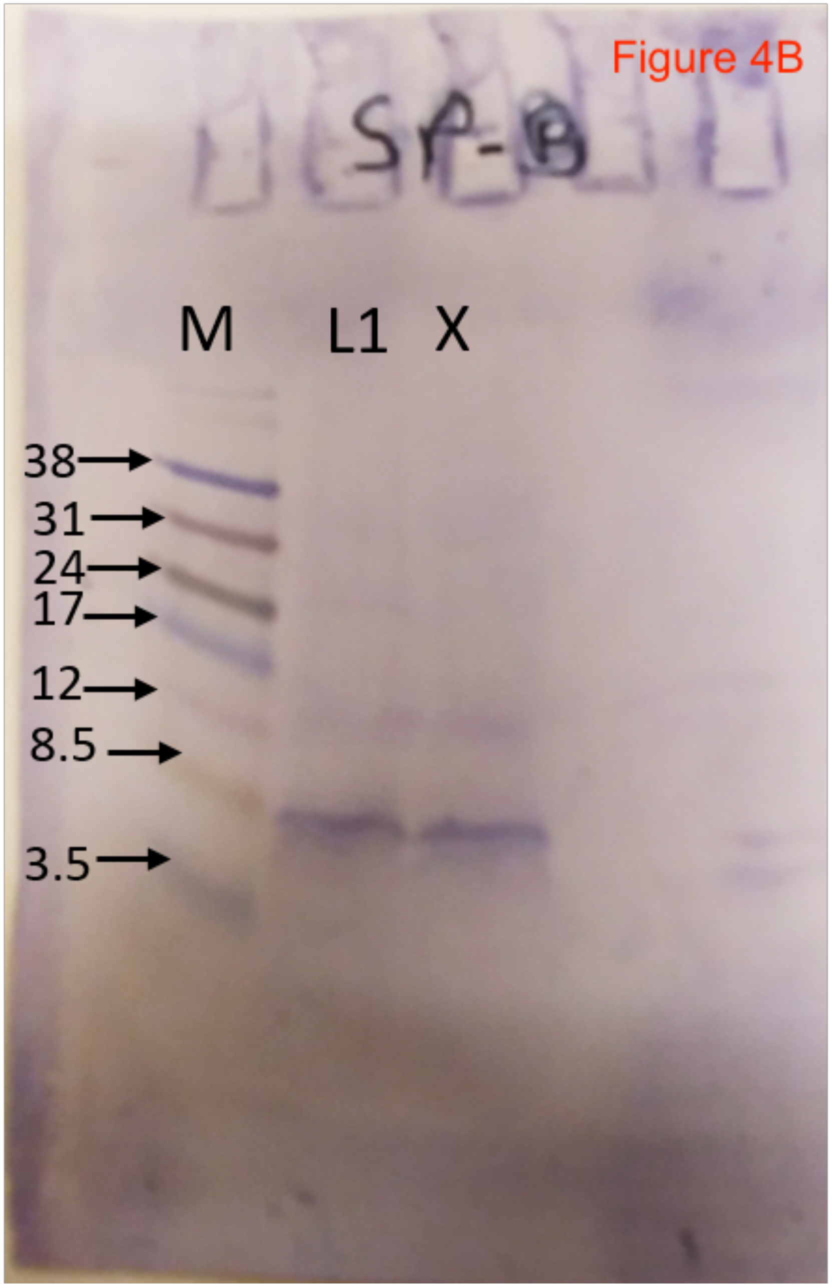

Blot; not duplicate of Fig 4A

M = marker

L1 = SP-B

X = duplicate sample as in L1 (cut off)
